# Gap junctions contribute to anchorage-independent clustering of breast cancer cells

**DOI:** 10.1186/s12885-018-4148-5

**Published:** 2018-02-27

**Authors:** Fabien Gava, Lise Rigal, Odile Mondesert, Elise Pesce, Bernard Ducommun, Valérie Lobjois

**Affiliations:** 1 Université de Toulouse, ITAV, CNRS, Toulouse, France; 2 CHU de Toulouse, Toulouse, France; 3 Centre Pierre Potier, ITAV-USR3505, 1 Place Pierre Potier, 31106 Toulouse Cedex, France

**Keywords:** Cancer cell clustering, Anchorage-independent aggregation, Gap junction intercellular communication

## Abstract

**Background:**

Cancer cell aggregation is a key process involved in the formation of clusters of circulating tumor cells. We previously reported that cell-cell adhesion proteins, such as E-cadherin, and desmosomal proteins are involved in cell aggregation to form clusters independently of cell migration or matrix adhesion. Here, we investigated the involvement of gap junction intercellular communication (GJIC) during anchorage-independent clustering of MCF7 breast adenocarcinoma cells.

**Methods:**

We used live cell image acquisition and analysis to monitor the kinetics of MCF7 cell clustering in the presence/absence of GJIC pharmacological inhibitors and to screen a LOPAC® bioactive compound library. We also used a calcein transfer assay and flow cytometry to evaluate GJIC involvement in cancer cell clustering.

**Results:**

We first demonstrated that functional GJIC are established in the early phase of cancer cell aggregation. We then showed that pharmacological inhibition of GJIC using tonabersat and meclofenamate delayed MCF7 cell clustering and reduced calcein transfer. We also found that brefeldin A, an inhibitor of vesicular trafficking, which we identified by screening a small compound library, and latrunculin A, an actin cytoskeleton-disrupting agent, both impaired MCF7 cell clustering and calcein transfer.

**Conclusions:**

Our results demonstrate that GJIC are involved from the earliest stages of anchorage-independent cancer cell aggregation. They also give insights into the regulatory mechanisms that could modulate the formation of clusters of circulating tumor cells.

**Electronic supplementary material:**

The online version of this article (10.1186/s12885-018-4148-5) contains supplementary material, which is available to authorized users.

## Background

Metastasis formation requires evasion of cancer cells from the primary tumor site, local invasion, intravasation into blood and lymphatic vessels, extravasation at distant sites and formation of metastatic deposits that will develop as metastatic lesions [1, 2]. Cancer cell dissemination and invasion have been prominently associated with the activation of the epithelial-to-mesenchymal transition (EMT) program. This program induces cancer cell morphological and motility changes and leads to the inhibition of the expression of cell-cell adhesion molecules [1]. However, in the absence of anchorage, tumor cells that have evaded from a primary tumor must develop adaptive strategies, such as cell aggregation and cluster formation, to prevent anoikis [3]. Therefore, an alternative model for cancer cell dispersion has been recently proposed. It involves the escape of clusters of aggregated cells from the primary tumor or single-cell clustering during dissemination, as suggested by the detection of clusters of circulating tumor cells (CTCs) in blood samples from metastatic patients [2, 4]. It has been shown that the formation of multicellular clusters prolongs cancer cell survival [5]. CTC clusters have 23- to 50-fold increased metastatic potential than isolated CTCs and are associated with adverse outcomes [4]. Circulating tumor microemboli (CTM), in which cancer cells are associated with platelets, stromal cells and hematopoietic cells, might also protect tumor cells from apoptosis [6]. Thus, CTC clusters greatly contribute to cancer cell metastatic spread.

Several years ago, it was shown that increased expression of E-cadherin, a major component of cell adherens junctions, enhances the formation of cell aggregates [7]. This is consistent with the finding that loss of functional E-cadherin is associated with cancer cell invasiveness and metastasis formation [1]. On the other hand, increased cancer cell adhesion properties also have been associated with higher experimental metastatic potential in vivo [8, 9].

Tumor cell aggregation regulation involves cell–cell adhesion proteins, including members of the cadherin superfamily [7], as well as other cell surface-associated proteins, such as MUC1 and galectin-3 [5]. In patients with breast cancer, the cell junction component plakoglobin is overexpressed compared with healthy tissue. In mouse models, plakoglobin knockdown abrogates CTC cluster formation and suppresses lung metastases [4]. High expression level of plakoglobin associated with cluster formation is in line with our in vitro data showing the regulatory role of desmosomal junctions on cancer cell aggregation [10]. Indeed, we previously developed a microscopy-based quantification assay to monitor the clustering kinetics of tumor cells in the absence of cell–substrate adhesion [10]. Using this approach, we showed that in colon and breast cancer cells, E-cadherins and also two desmosomal proteins (desmoglein and desmocolin) contribute to cancer cell aggregation.

Gap junctions connect two adjacent cells by establishing the continuity of connexons formed by a hexamer of subunits called connexins. Gap junction activity is associated with their ability to generate gap junction intercellular communication (GJIC) and this can be monitored with cellular assays based on the transfer of fluorescent probes between cells [11–13]. Connexin may also have a role in autocrine or paracrine diffusion when hemi-channels are active between the cytoplasm and the extracellular medium. Gap junctions are also involved in different signaling pathways, particularly through the interaction of the C-terminal domains of connexins with several proteins (reviewed in [14]), including cytoskeleton proteins (directly with tubulin [15] and indirectly with actin [16]). Several studies have shown that gap junction formation and maintenance require both vesicular transport [17] and the actin cytoskeleton, which could provide tracks and specificity for membrane delivery [18].

Here, we examined the role of GJIC in the anchorage-independent clustering of MCF7 cancer cells. Using GJIC pharmacological inhibitors, we demonstrated their involvement during the early step of anchorage-independent cancer cell clustering. Inhibition of the actin cytoskeleton using latrunculin A and of vesicular trafficking using brefeldin A, which we identified in a small compound library screening, resulted in GJIC and cell clustering inhibition, in agreement with the requirement of the actin cytoskeleton and vesicular trafficking for GJIC formation. Altogether, these data reinforce the idea that GJIC are potential key players in the early steps of the metastatic process, and could be used to identify new strategies for therapeutic intervention.

## Methods

### Cell culture

MCF7 cells (ATCC HTB-22) were cultured in RPMI (Gibco, Life Technologies) supplemented with 1 μmol/L insulin (Sigma Aldrich), 10% fetal calf serum (FCS) (Gibco, Life Technologies) and 1% penicillin/streptomycin (100 U/mL, Gibco, Life Technologies) in a humidified atmosphere of 5% CO2 at 37 °C.

### Chemicals and reagents

Meclofenamate (10 mM in water), tonabersat (40 mM in DMSO), latrunculin A (1 mM in DMSO), brefeldin A (10 mM in DMSO), and calcein AM (4 mM in DMSO), were purchased from Sigma Aldrich.

### Cell clustering assay

The cell clustering assay used in this study was adapted with slight modifications from the one described in our previous report [10]. Specifically, DMEM-F12 (Gibco, Life technologies) supplemented with 1 μmol/L insulin, 10 nmol/L beta-estradiol, 20 ng/mL epidermal growth factor (Invitrogen), B-27 Supplement (1X, Invitrogen) and 1% penicillin/streptomycin (100 U/mL, Gibco, Life technologies) was used as medium. MCF7 cells were harvested at 70-80% confluence from T25 flasks using trypsin (0.05% trypsin- 0.53 mM EDTA; Gibco, Life Technologies) and counted. 500 cells in 50 μl medium per well were distributed in Greiner 96-well round bottom plates, except in the 36 peripheral wells to avoid edge effects (thus, 60 exploitable wells/plate). Cells were centrifuged (200 g for 4 min), and then the drug of interest, diluted in the same medium (2X the desired concentration, 50 μL per well), or medium only (non-treated condition) was added, followed by another centrifugation step. Plates were then placed in a humidified atmosphere with 5% CO_2_ at 37 °C and processed for microscopy.

### Microscopy, image processing and quantification

Based on our previous study [10], cell clustering in each well was followed by time-lapse video-microscopy. Images were acquired with an inverted wide-field Zeiss Z1 Observer Axio microscope, using a 0.3 N.A 10X objective and a CoolSNAP CDD camera (Roper scientific) in bright-field (transmitted light) for at least 10 h with one acquisition every 15 min. At each time point and position, 10-μm spaced z-stacks over 100 μm depth (11 stacks) in bright-field were acquired using the MetaMorph software*.* The main steps of the algorithm to monitor and measure the cell clusters over time with a custom-made MATLAB procedure were: (1) at each time point, and for each cluster, images were processed by focus stacking to merge images of multiple focal planes into one in-focus image (with ImageJ), (2) binarization and edge detection with a Sobel filter were used to define the cluster boundaries, as well as the boundaries of holes inside clusters to exclude them, (3) saving of the projection, segmentation and image overlay, and (4) calculation of the typical parameters (perimeter, area, normalized area: Area T0/Area T(x)). Results are presented as the Normalized area reduction over time.

### Gap junction intercellular communication assay and flow cytometry

This assay was performed in the same experimental setting as described above. After incubation with 0.1 μM calcein AM (cell-permeant stain; 30 min of incubation in 5% CO_2_ at 37 °C in T25 flasks), 50% of stained cells were mixed with 50% of unlabeled cells before distribution in wells. Cells were retrieved at 0, 2, 5 and 10 h after the beginning of the assay. Cells from 10 wells for the same condition were pooled to obtain one replicate/sample, allowing to obtain three (half plate) or six replicates (an entire plate) per condition for each independent experiment. Clusters were dissociated (mechanically and with trypsin) in single-cell suspensions and rinsed (1X PBS) before flow cytometry (BD C6 Accuri) analysis of calcein green fluorescence.

### Double labeling dye transfer

The experimental procedure was identical to that of the GJIC assay described above, with the exception that cells were stained with calcein AM together with HCS Cell Mask Deep Red (4 μg/mL, Life Technologies), which does not transit through gap junctions.

### Immunofluorescent staining

Cells grown on coverslips for 3 days were washed in PBS and fixed in formalin for 10 min. After washes and permeabilization in PBS containing 0.5% Triton X-100 at room temperature (RT) for 5 min, cells were incubated in PBS containing 1% BSA at RT for 1 h. Then, they were incubated at 4 °C with antibodies against connexin CX43 (1/100, Cell Signaling #3512) in PBS/1% BSA overnight. After washes in PBS/0.1% Triton X-100, goat anti-rabbit Alexa 488 antibodies (Molecular Probes, 1/500) were added at RT for 1.5 h.

### Screening of the compound library and hit characterization

The LOPAC® commercial library (1280 compounds) from Sigma-Aldrich was used for this screen. The screening strategy was to search for compounds that inhibit cell aggregation at the unique concentration of 1.25 μM. EDTA was used as positive control to calculate the Z’ factor (> 0.7) and to validate each library batch. 500 MCF7 cells per well were distributed in 96-well round bottom plates (Greiner). Plates were centrifuged (200 g for × 8 min) and then placed in a humidified atmosphere of 5% CO_2_ at 37 °C on the stage of the video-microscope to monitor cell aggregation. Images were acquired at the time 0 and during 5 h. 5 μm spaced z-stacks over 100 μm depth (21 stacks) in bright-field were acquired using the MetaMorph software. Images were processed as described above. The normalized area reduction over time was the assessment criterion. Molecules that reduced cell aggregation were then validated with a dose-response test using six replicates per concentration, with images acquired every 15 min for 10 h.

### Software

The BD Accuri software was used for flow cytometry data analysis and description of the results, and GraphPad Prism for graph conception.

### Statistical analysis

For statistical analyses, the GraphPad Prism software was used. The normal distribution of data was assessed with the Kolmogorov-Smirnov, D’Agostino & Pearson, and Shapiro-Wilk tests. Homoscedasticity was also checked and if variances were significantly different, statistical tests were performed with Welch’s correction; ***: *p* < 0.0005, **: *p* < 0.005, *: *p* < 0.05 for all figures.

## Results

### Functional gap junctions are established during clustering of MCF7 cancer cells

**Fig. 1:**
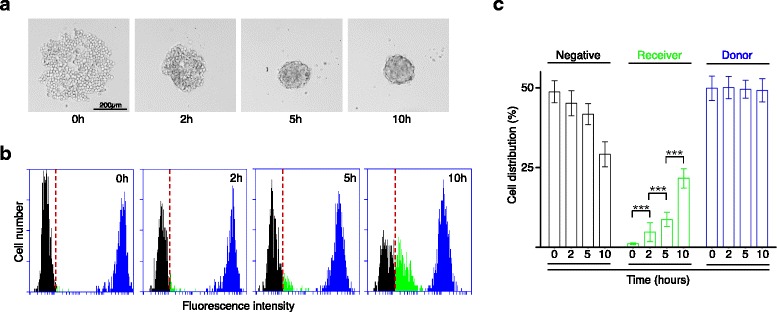
Functional GJIC is established during clustering of MCF7 cancer cells. **a** Clustering of MCF7 cells in the absence of anchorage. Representative transmitted light microscopy images of the clustering assay at the indicated time points. **b**, **c** In experimental conditions identical to (**a**), analysis of fluorescent dye transfer between calcein-loaded cells (blue) and negative cells (black). (**b**) Flow cytometry quantification of fluorescence intensity. Newly positive cells (fluorescence intensity higher than the threshold indicated by the dotted red vertical line) are identified as receiver cells (green). **c** Quantification of the flow cytometry analysis. At each time point of the experiment, the percentage of cells in each category is indicated. Results are the mean ± SD of 4 independent experiments with at least 3 replicates per conditions. Unpaired two-tailed t-tests, *** *p* < 0.0005

As already published [10] and illustrated in Fig. 1a, when seeded in anchorage-free conditions that prevent cell adhesion to the substrate, breast adenocarcinoma MCF7 cells progressively clustered to form a solid shaped aggregate within 5 h. This assay allows the accurate and reproducible quantification of cancer cell clustering and was previously used to demonstrate the role of E-cadherin and desmosomal proteins in this process [10].

Here, we used this assay to determine whether functional GJIC was established during cell clustering. To this aim, we adapted a classical dye transfer assay based on loading part of the cell population with the fluorescent dye calcein and on monitoring its transfer from loaded donor cells to receiver cells (see Methods). After mixing calcein-positive and –negative MCF7 cells at the time 0 of the clustering assay, we monitored by flow cytometry analysis calcein fluorescence level in the population over time (Fig. 1b). We detected calcein-positive (blue) and calcein-negative (black) cells at the start of the experiment. During the experiment, receiver cells with intermediate fluorescence intensity (green) progressively appeared and increased in number, while concomitantly the percentage of the negative population decreased. Quantification of these data (Fig. 1c) clearly showed that the percentage of receiver cells (mean ± SD) progressively and significantly increased: 4.7% (±2.9) at 2 h, 8.7% (±2.3) at 5 h and 21.6% (±3.1) at 10 h. To confirm that the observed transfer was due to GJIC, we performed control experiments in which donor cells were double labeled with calcein and the non-diffusible HCS Cell Mask Deep Red dye (see Additional file 1: Figure S1).

**Fig. 2:**
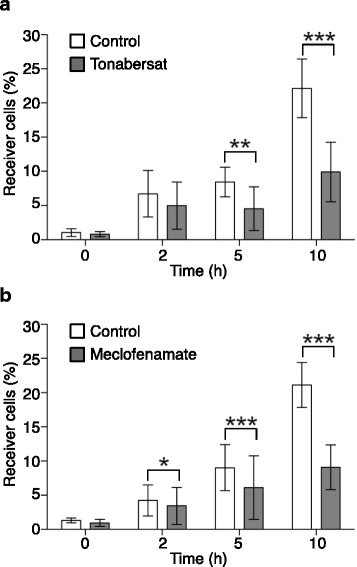
Pharmacological inhibition of GJIC during MCF7 cancer cell clustering. Effect of incubation with (**a**) tonabersat (300 μM) and (**b**) meclofenamate (300 μM) versus control (not treated) on calcein transfer from donor positive cells to negative cells during the clustering assay. The percentage of receiver cells (negative cells that become calcein-positive) is indicated. Results are the mean ± SD of 4 independent experiments for tonabersat and 3 independent experiments for meclofenamate (3 replicates per condition in each experiment). Unpaired two-tailed t-tests, except for (**b**) at 10 h: Mann-Whitney non-parametric test, *p < 0.05, **p < 0.005 and ***p < 0.0005

To further confirm our observation, we next examined the effect of two widely used GJIC inhibitors (tonabersat [19] and meclofenamate [20]) on calcein dye transfer during clustering. Incubation with tonabersat (Fig. 2a) or with meclofenamate (Fig. 2b) resulted in a similar, significant reduction of the number of receiver cells over time compared with untreated cells (control).

Altogether these observations indicate that the formation of functionally active GJIC between MCF7 cells is a rapid process that occurs within few hours during aggregation of cells that are placed in anchorage-free conditions.

### Gap junction pharmacological inhibition impairs MCF7 cell clustering

**Fig. 3:**
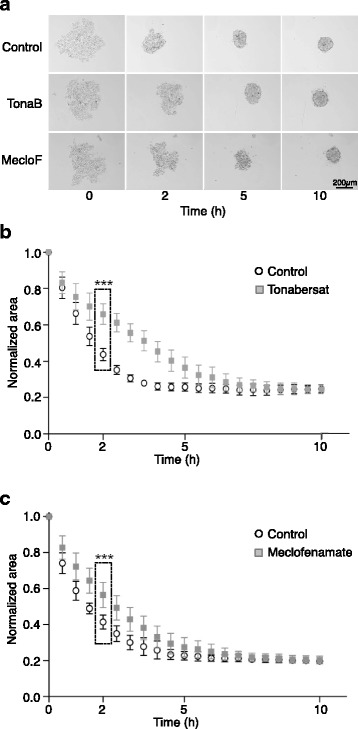
Effect of GJIC pharmacological inhibition on MCF7 cancer cell clustering. **a** Representative in-focus bright-field images are shown for each experimental condition at the indicated time points. **b**, **c** Variation of the area occupied by MCF7 cells during the clustering assay with cells incubated with: (**b**) tonabersat (300 μM, *n* = 17) or not (control) (*n* = 14), or (**c**) meclofenamate (300 μM, *n* = 16) or not (control) (*n* = 16). At 2 h, *p* < 0.0001. Results are the mean ± SD of 3 independent experiments. Unpaired two-tailed t-tests at 2 h, ***p < 0.005

These observations prompted us to examine whether the early stages of GJIC formation play an active role in cancer cell clustering. To this aim, we evaluated the effect of GJIC pharmacological inhibition with tonabersat and meclofenamate on anchorage-independent MCF7 cell clustering by monitoring cell clustering at the beginning of the assay (T0) and after 2, 5 and 10 h (Fig. 3a). Control (untreated) cells initially formed a slightly irregular non-adherent monolayer disk, and then progressively gathered together and aggregated to form a small 3D cluster. Both tonabersat and meclofenamate slightly delayed early clustering of MCF7 cells. We then quantified cell aggregation by determining the cluster surface area relative to T0 using a dedicated image analysis tool developed in MATLAB ([10] and see Methods). This quantification showed that the clustering area at 2 h was reduced by 59% in control condition, and by 34% and 43% upon GJIC pharmacological inhibition with tonabersat and meclofenamate, respectively (*p* < 0.0005 vs control for both inhibitors) (Fig. 3b and c). This quantification confirmed that the two inhibitors significantly slowed down cell clustering at the early stage, but that at the end of the clustering assay (10 h), the normalized area reduction was comparable in treated and untreated samples.

Together these data led to the conclusion that functionally active gap junctions could participate and contribute to the early phase of MCF7 breast cancer cell clustering in anchorage-independent conditions.

### Actin cytoskeleton is required for functional GJIC and for MCF7 cell clustering

**Fig. 4:**
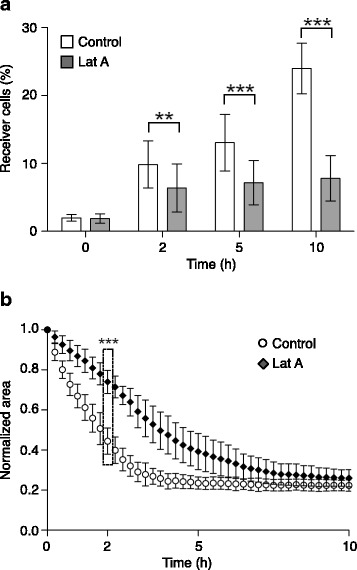
Effect of latrunculin A on MCF7 cancer cell clustering and calcein transfer. **a** Transfer of calcein during clustering from donor positive cells to negative cells incubated or not with 100 nM latrunculin A. The percentage of receiver newly positive cells is indicated. Results are the mean ± SD of 7 independent experiments. Unpaired two-tailed t-tests, **p < 0.005 at 2 h, and ***p < 0.0005 at 5 h and 10 h. **b** Variation of the area occupied by MCF7 cells during the clustering assay with cells incubated or not with 100 nM latrunculin A (*n* = 22). Results are the mean ± SD of 4 independent experiments. Unpaired two-tailed t-tests at 2 h, ***p < 0.0005

Actin cytoskeleton disruption using latrunculin A results in the alteration of GJIC localization at the cell membrane [18]. Therefore, we examined whether this might also result in MCF7 cell clustering inhibition. We first confirmed that incubation of MCF7 cells with 100 nM latrunculin A very significantly inhibited the progressive increase in the percentage of receiver (newly calcein-positive) cells in our GJIC assay (Fig. 4a). The extent of this inhibition was comparable to what observed with the two GJIC pharmacological inhibitors (see Fig. 2). Moreover, latrunculin A incubation also significantly slowed down cell clustering (*p* < 0.0005), as indicated by the slower reduction of the normalized area during the first hours of the assay (Fig. 4b). However, after 5 h this effect was reduced and, at 10 h, cell clustering was comparable in treated and control cells. Finally, incubation with both latrunculin A and meclofenamate did not have any additive effect on calcein transfer inhibition, while it had an additional effect on the inhibition of cell clustering (Additional file 2: Figure S2).

These data indicate that actin cytoskeleton integrity, which is needed for functional GJIC, is also required during the early phase of MCF7 cell clustering.

### Inhibition of vesicular transport using brefeldin a results in the inhibition of GJIC function and MCF7 cell clustering

**Fig. 5:**
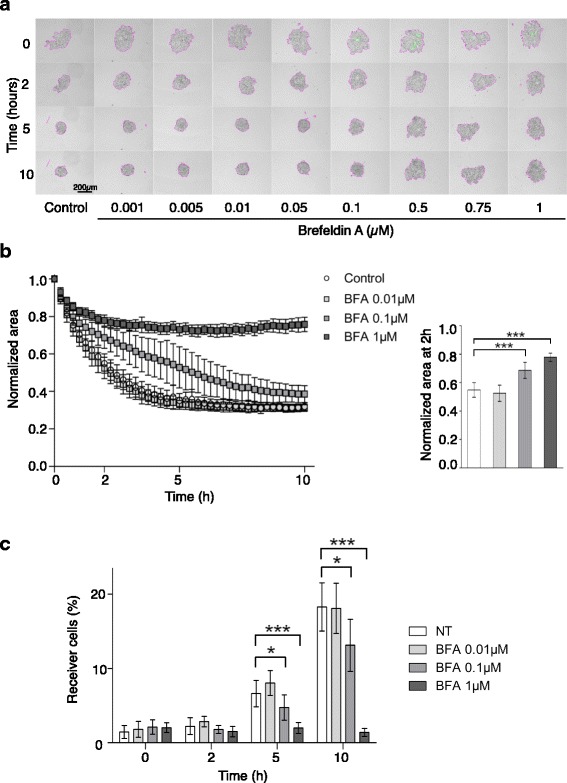
Brefeldin A inhibits MCF7 cancer cell clustering and calcein transfer. **a** Representative in-focus bright-field images are shown for each brefeldin A concentration and at the indicated time points. **b** Variation of the area occupied by MCF7 cells during the clustering assay with cells incubated with brefeldin A at the following concentration: 0.01 μM (*n* = 10), 0.1 μM (*n* = 17), 1 μM (*n* = 16) compared with untreated Control (*n* = 17). Results are the mean ± SD of 3 independent experiments (except for 0.01 μM: 2 independent experiments). Unpaired two-tailed t-tests at 2 h, ***p < 0.0005. **c** Transfer of calcein during clustering from donor positive cells to negative cells incubated or not with 0.01, 0.1, and 1 μM brefeldin A. The percentage of receiver newly positive cells is indicated. Results are the mean ± SD of 3 independent experiments (3 replicates per experiment). Unpaired two-tailed t-tests, at 5 h and 10 h *p < 0.05 and ***p < 0.0005

To find new regulators and signaling pathways involved in MCF7 cell clustering in anchorage-independent conditions, we developed a screening strategy based on our cell aggregation assay. We used the LOPAC® commercial chemical library of 1280 small bioactive compounds to search for cell aggregation inhibitors at the 5 h end point. Amongst several hits (ongoing work), we focused on brefeldin A. Upon treatment with brefeldin A, MCF7 cell clustering was strongly reduced in a concentration-dependent manner and was totally inhibited at the 0.5 μM concentration (Fig. 5a). Quantification of these results confirmed that cell clustering inhibition was concentration-dependent and that this inhibition was significant (at 0.1 μM) and complete when using 1 μM brefeldin A (Fig. 5b).

It has been reported that gap junction formation and maintenance are dependent on vesicular transport [17]. Therefore, we tested in our assay whether brefeldin A impaired calcein transfer. We observed no effect with 0.01 μM brefeldin A. Conversely, 0.1 μM brefeldin A reduced the progressive increase of the percentage of receiver cells at 5 and 10 h compared with control (untreated, NT), and 1 μM brefeldin A completely inhibited calcein transfer (Fig. 5c). In addition, immunofluorescence analysis of connexin CX43 expression revealed loss of cytoplasmic membrane localization upon treatment with 1 μM brefeldin A (Additional file 3: Figure S3).

These data indicate that pharmacological inhibition of vesicular transport leads to impaired delivery of GJIC to the membrane and alteration of calcein transfer via functional GJIC, resulting in the inhibition of MCF7 cell clustering.

## Discussion

How tumor cell clustering contributes to cancer progression and how it is regulated remain unclear. Loss of E-cadherin, a master cell-cell adhesion protein, is an EMT hallmark associated with metastasis. However, the detection of clusters of CTCs that overexpress cell-cell adhesion proteins in patients with cancer has been correlated with high metastatic potential and poor prognosis. These observations suggest pro- and anti-tumor roles for cell aggregation and strengthen the need to precisely understand the regulatory mechanisms and how variations in the cancer cell cluster formation potential could contribute to cancer progression.

In this work, we investigated GJIC role in the sequence of events leading to breast cancer cell cluster formation. We adapted and used a classical calcein transfer assay to demonstrate that very rapidly after seeding in anchorage-independent conditions, GJIC are formed between MCF7 cells (Fig. 1). In addition, we confirmed the specificity of this assay using the non-diffusible HCS Cell Mask Deep Red dye (Additional file 1: Figure S1).

We also used tonabersat and meclofenamate, two reference compounds that reduce metastasis progression thanks to their gap junction inhibitor function [21], to inhibit GJIC-dependent calcein transfer (Fig. 2). We observed that these compounds also slowed down cell clustering (Fig. 3). This inhibitory effect was obvious and maximum around 2-3 h after seeding (i.e., the very early stage of GJIC formation), suggesting that adhesiveness of the hemi-connexons might be sufficient to contribute to cell adhesion. Of course, adherens junctions and desmosomes [10, 22] are also rapidly engaged in the adhesion process, and might compensate for the lack of functional GJIC, which explains why the effect on cell clustering of GJIC pharmacological inhibition progressively decreases and is lost after 5 h.

To further demonstrate that GJIC plays a key role at a very early stage of cell clustering, we manipulated its formation and assembly at the cell membrane. It is well documented that inhibition of the actin cytoskeleton dynamics with latrunculin A affects GJIC formation [18]. Using latrunculin A, we again observed inhibition of MCF7 cell clustering that was maximal at 2 h and was resumed after 5 h (Fig. 4). Furthermore, the absence of additive effect on calcein transfer inhibition with latrunculin A and meclofenamate suggest that these compounds act on the same mechanism. Although we cannot exclude that another actin cytoskeleton-dependent early event could participate in driving MCF7 cell clustering, these observations strongly reinforce the conclusion of a key and initiating role for GJIC.

With the aim to identify novel pathways involved in the regulation of anchorage-independent MCF7 cell clustering, we performed a pharmacological screen of a commercial bioactive small-compound library. We did not identify cytoskeleton inhibitors in this screen, probably because the selected endpoint was 5 h, when latrunculin A effect is already compensated. However, together with other hits that are currently under investigation, we identified brefeldin A as a potent inhibitor of anchorage-independent MCF7 cell clustering (Fig. 5). Moreover, we observed that in the same experimental setting, brefeldin A also inhibited calcein transfer. This is fully in agreement with previous reports indicating that vesicular trafficking is required for GJIC formation and maintenance [17], which is also confirmed by the loss of connexin CX43 membrane localization upon incubation with brefeldin A (Additional file 3: Figure S3). However, in contrast to what we observed using GJIC inhibitors or latrunculin A, brefeldin A inhibitory effect on cell aggregation did not stop after 5 h, suggesting that other important mechanisms required during clustering are also dependent on vesicular trafficking.

## Conclusions

Altogether the results reported in this study reinforce the importance of considering GJIC as potential key players in the early steps of the metastatic process, with the aim to decipher the mechanisms that lead to cancer cell-cell adhesion and cluster formation, and to identify new strategies for therapeutic intervention.

## Additional files

Additional file 1: **Figure S1.** Specificity of calcein dye transfer. To ensure the GJIC specificity of the observed calcein transfer, control experiments were performed in which cells were loaded with calcein together with the non- diffusible HCS Cell Mask Deep Red dye. The dye transfer was quantified by flow cytometry at time 0 and after 10 h, both in control condition (only co-staining) and in co-labeled cells incubated with the GJIC inhibitor meclofenamate. The calcein and Cell Mask negative cell population progressively became positive for calcein in the control condition, but not in cells incubated with meclofenamate. Conversely, no transfer of the Cell Mask dye to negative cells was observed in control and meclofenamate-treated cells. (PDF 38 kb)

Additional file 2: **Figure S2.** Effect of the combination of latrunculin A and meclofenamate on the clustering of MCF7 cancer cells and on calcein transfer. (A) Variation of the area occupied by MCF7 cells during the clustering assay with cells incubated or not (*n* = 21) with 100 nM latrunculin A (*n* = 22), 300 μM meclofenamate (*n* = 26), or latrunculin A + meclofenamate (*n* = 27). Results are the mean ± SD of 4 independent experiments. Mann-Whitney non-parametric tests, except for NT versus latrunculin A + meclofenamate: unpaired two-tailed t-test at 2 h, ***p < 0.0005. (B) Transfer of calcein during the clustering assay from donor positive cells to negative cells incubated or not with different compounds as in (A). The percentage of receiver positive cells is indicated. Results are the mean ± SD of 4 independent experiments (3 replicates for each condition in each experiment). Unpaired two-tailed t-tests, at 2 h, 5 h and 10 h; differences are not statistically significant (N.S.). (PDF 575 kb)

Additional file 3: **Figure S3.** Characterization of Cx43 localization in MCF7 cells incubated or not with brefeldin A and latrunculin A. MCF7 cells were incubated with brefeldin A (1 μM) and latrunculin A (100 nM and 400 nM), or not, for 5 h. Cx43 expression is in green, DAPI staining of nuclei in blue. Magnification: 40X, scale bar: 20 μm. (PDF 2439 kb)

## Abbreviations

CTC: Circulating Tumor cells
CTM: Circulating Tumor Microemboli
EMT: Epithelial to Mesenchymal Transition
GJIC: Gap Junction Intercellular Communication
IC50: Inhibitory Concentration 50%
