# Supplementary figures and images for: Gap junctions contribute to anchorage-independent clustering of breast cancer cells
Source: BMC Cancer. 2018 Feb 27;18:221. doi: 10.1186/s12885-018-4148-5 (PMC5828067; doi:10.1186/s12885-018-4148-5)

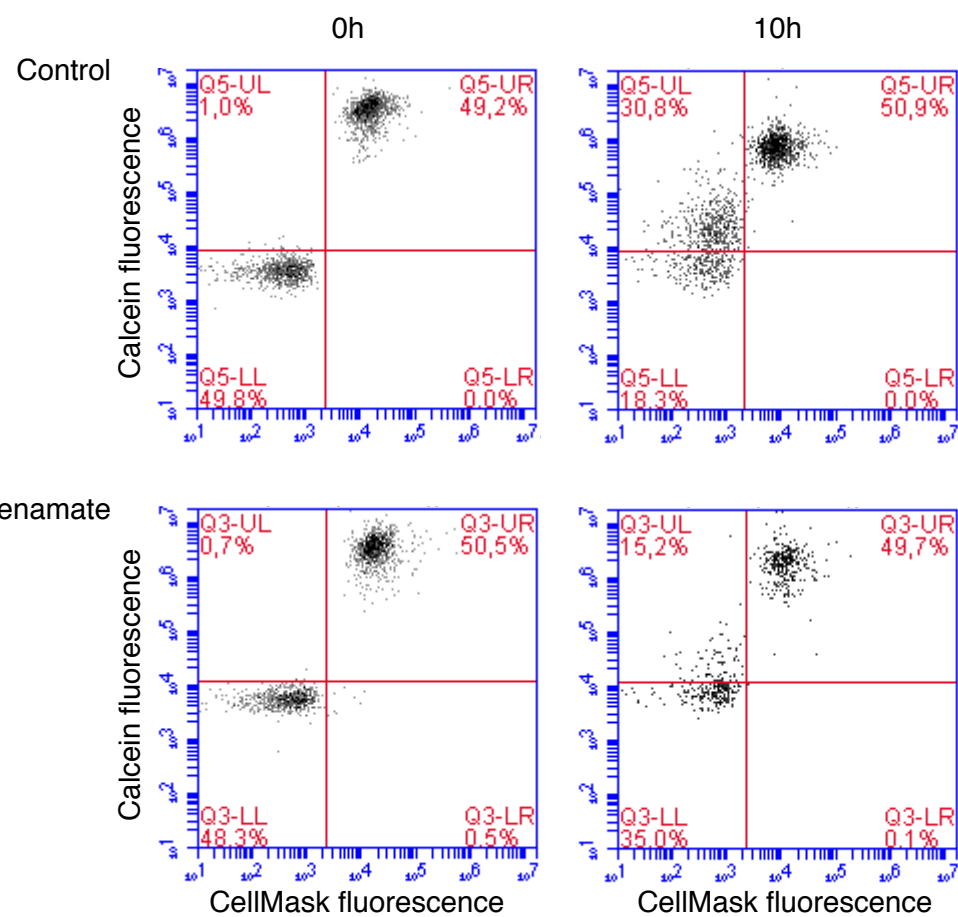

Supplement: Supplementary file 1 — Figure S1. Specificity of calcein dye transfer. To ensure the GJIC specificity of the observed calcein transfer, control experiments were performed in which cells were loaded with calcein together with the non- diffusible HCS Cell Mask Deep Red dye. The dye transfer was quantified by flow cytometry at time 0 and after 10 h, both in control condition (only co-staining) and in co-labeled cells incubated with the GJIC inhibitor meclofenamate. The calcein and Cell Mask negative cell population progressively became positive for calcein in the control condition, but not in cells incubated with meclofenamate. Conversely, no transfer of the Cell Mask dye to negative cells was observed in control and meclofenamate-treated cells. (PDF 38 kb) [file 12885_2018_4148_MOESM1_ESM.pdf]

A

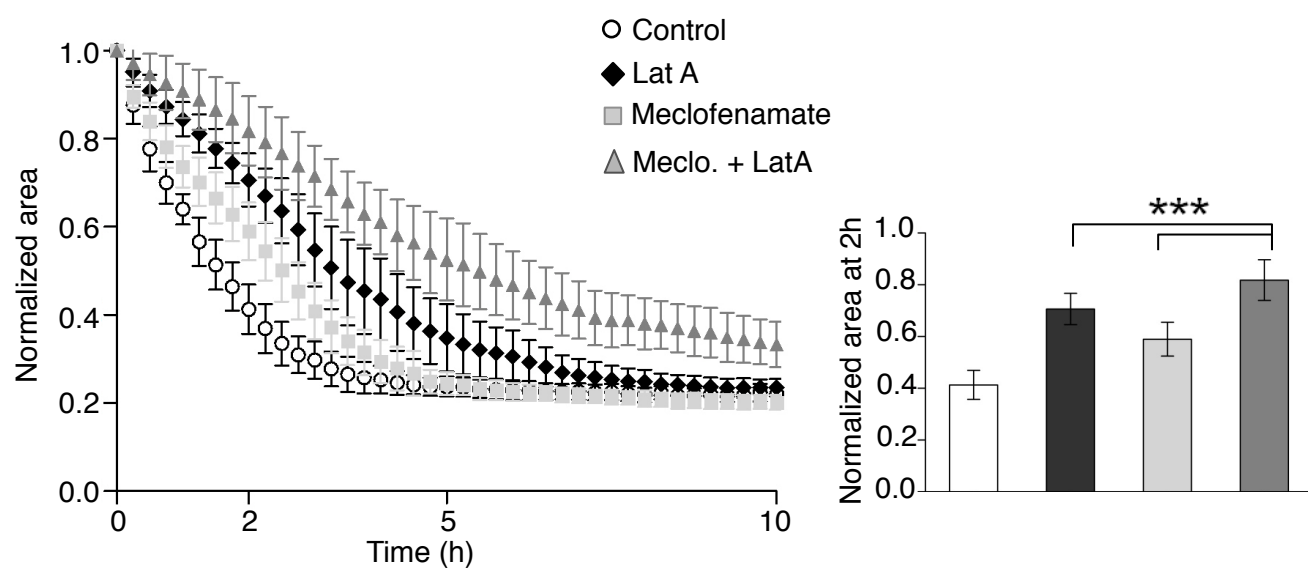

B

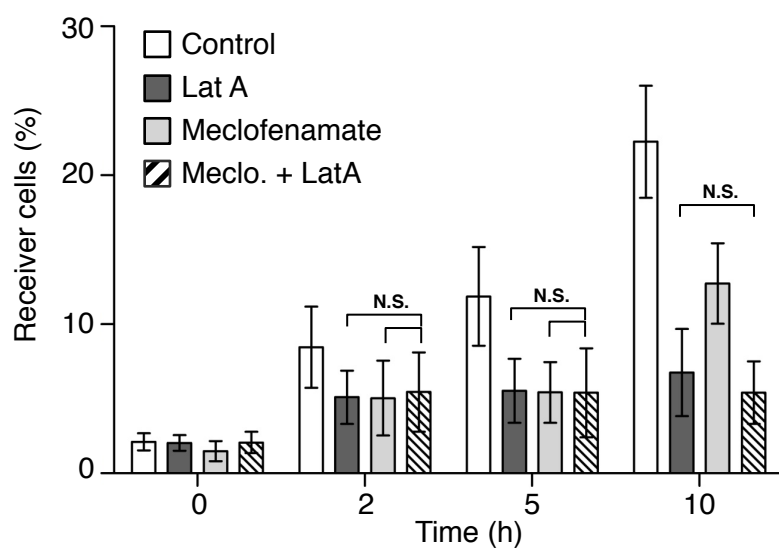

Supplement: Supplementary file 2 — Figure S2. Effect of the combination of latrunculin A and meclofenamate on the clustering of MCF7 cancer cells and on calcein transfer. (A) Variation of the area occupied by MCF7 cells during the clustering assay with cells incubated or not (n = 21) with 100 nM latrunculin A (n = 22), 300 μM meclofenamate (n = 26), or latrunculin A + meclofenamate (n = 27). Results are the mean ± SD of 4 independent experiments. Mann-Whitney non-parametric tests, except for NT versus latrunculin A + meclofenamate: unpaired two-tailed t-test at 2 h, ***p < 0.0005. (B) Transfer of calcein during the clustering assay from donor positive cells to negative cells incubated or not with different compounds as in (A). The percentage of receiver positive cells is indicated. Results are the mean ± SD of 4 independent experiments (3 replicates for each condition in each experiment). Unpaired two-tailed t-tests, at 2 h, 5 h and 10 h; differences are not statistically significant (N.S.). (PDF 575 kb) [file 12885_2018_4148_MOESM2_ESM.pdf]

Untreated

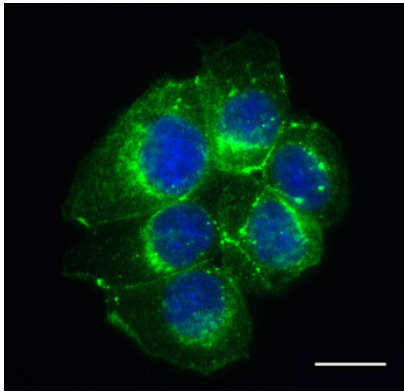

Brefeldin A 1 $\mu$ M

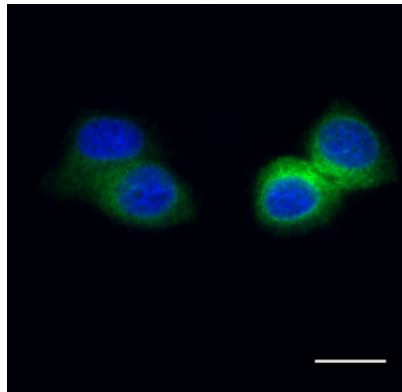

Latrunculin A

---

100nM

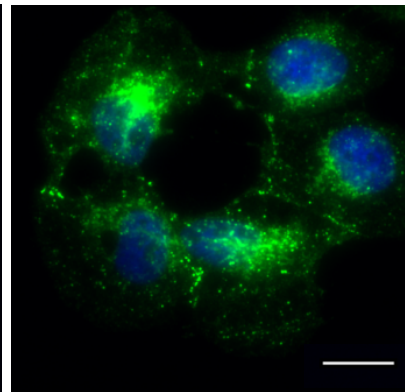

400nM

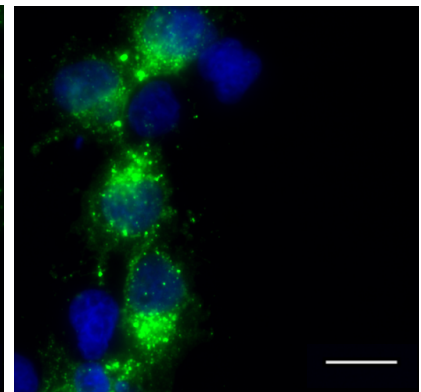

Supplement: Supplementary file 3 — Figure S3. Characterization of Cx43 localization in MCF7 cells incubated or not with brefeldin A and latrunculin A. MCF7 cells were incubated with brefeldin A (1 μM) and latrunculin A (100 nM and 400 nM), or not, for 5 h. Cx43 expression is in green, DAPI staining of nuclei in blue. Magnification: 40X, scale bar: 20 μm. (PDF 2439 kb) [file 12885_2018_4148_MOESM3_ESM.pdf]
